# Housing and youth mental health during a COVID-19 lockdown

**DOI:** 10.1192/j.eurpsy.2021.1938

**Published:** 2021-08-13

**Authors:** J. Groot, A. Keller, A. Joensen, T.-L. Nguyen, A.-M. Nybo Andersen, K. Strandberg-Larsen

**Affiliations:** 1 Department Of Public Health, University of Denmark, Copenhagen K, Denmark; 2 Section Of Epidemiology, University of Copenhagen, Copenhagen, Denmark

**Keywords:** public health, mental health, COVID-19, housing

## Abstract

**Introduction:**

Declines in mental health among youth in the COVID-19 pandemic have been observed, yet longitudinal studies on how housing may impact these declines are lacking.

**Objectives:**

Our aim was to determine whether changes in mental health among Danish youth were dependent on their housing conditions.

**Methods:**

Young participants from the Danish National Birth Cohort, who had responded to an online questionnaire at 18 years of age, and later during the initial national Danish lockdown, were included. Associations between housing conditions (direct access to outdoor spaces, urbanicity, household density, and household composition) and changes in mental health (mental well-being, quality of life (QoL) and loneliness) were examined in multivariate linear and logistic regression analyses.

**Results:**

We included 7455 participants. Greater decreases in mental well-being were observed for youth with no access to direct outdoor spaces and those living in denser households (mean difference -0.83 [95 % CI -1.19, -0.48], -0.30 [-0.43, -0.18], respectively). Onset of low mental well-being was associated with no access and living alone (odds ratios (OR) 1.68 [1.15, 2.47] and OR 1.47 [1.05, 2.07], respectively). Household density was negatively associated with QoL (mean difference -0.21 [-0.30, -0.12]). Youth living alone experienced more loneliness (OR 2.12 [95 % CI 1.59, 2.82]).

**Conclusions:**

How youth’s mental health changed from before to during lockdown was associated with housing conditions. Among the Danish youth in our study, greater decreases in mental health during lockdown were observed among youth without access to outdoor spaces, living alone, or living in denser households.

**Disclosure:**

No significant relationships.

